# *Actinidia arguta*: Biological and Health Promoting Properties—Analysis of Bioactive Components

**DOI:** 10.3390/plants14233565

**Published:** 2025-11-21

**Authors:** Irena Maria Choma, Małgorzata Olszowy-Tomczyk

**Affiliations:** Department of Chromatography, Institute of Chemical Sciences, Faculty of Chemistry, Maria Curie-Skłodowska University, Maria Curie-Skłodowska Sq. 3, 20-031 Lublin, Poland

**Keywords:** *Actinidia arguta*, mini kiwi, hardy kiwi, *Actinidiaceae*, bioactive components, chromatographic analysis, biological properties

## Abstract

*Actinidia arguta*, also known as mini kiwi (due to its small size) or hardy kiwi (due to its frost resistance), is becoming an increasingly popular fruit alongside its commercially older siblings, i.e., *A. deliciosa* (green kiwi fruit) and *A. chinensis* (golden kiwifruit), from the *Actinidiaceae* family. This review paper discusses the biological and pharmacological properties of *A. arguta* fruits, with a special focus on methods of the bioactive component analysis. Mini kiwi is a valuable source of bioactive compounds, which contribute to its health-promoting properties, among others: antioxidant, neuroprotective, anticholinergic, antitumor, anti-inflammatory, antidiabetic, antiobesity as well as antiatherosclerotic ones. They are briefly discussed, illustrating the action of bioactive ingredients and the methods of analysis, which are presented in the tables. This review includes a concise characterization of *A. arguta* and updates the current field of knowledge about its diverse biological activities, which are undoubtedly related to the content of bioactive components and the methods used for their isolation and analysis. The information included in this review paper will be helpful in perceiving mini kiwi not only as a tasty fruit but also as a source of bioactive ingredients with beneficial, health-promoting effects on the body. Effective isolation of these components can contribute to the future development of antiaging and anticancer drugs, which undoubtedly will lead to further research and promote this species.

## 1. Introduction

*A. arguta* (hardy kiwi, mini kiwi) belongs to the *Actinidiaceae* family, the genus *Actinidia*, together with *A. deliciosa* (*A. chinensis* var. *deliciosa*, green kiwifruit) and *A. chinensis* (*A. chinensis* var. *chinensis*, golden kiwifruit), which are very popular on the world market [1]. All species are perennial, woody climbing vines (lianas), growing naturally in woodlands of East Asia [2]. The popularity of *Actinidia* fruits is linked to their flavor and health benefits. They have had a long history of wild harvesting, while the process of domestication lasted over 100 years [3,4].

The cultivation of *A. chinensis* began in New Zealand in the early 20th century from seeds brought from China by the teacher Isabel Fraser [5]. It appeared commercially in the 1960s. Since then, it has become widespread and available year-round. At first, it was called “Chinese gooseberry” due to its sweet-spicy flavor and green color. This name did not gain popularity, and in 1959 it was finally changed to kiwi, after New Zealand’s national bird [6]. The diversity of wild *Actinidia* species has contributed to the creation of new varieties with many desirable features appreciated by customers and known for various morphological characteristics [7]. The *Actinidia* plants are cultivated commercially in New Zealand, Italy, Spain, Greece, France, the United States, Japan, Israel and Chile [8].

*A. arguta* is a tall climbing vine first described in 1843 by Philipp Franz von Siebold and Joseph Gerhard Zuccarini. The most recognized variety of hardy kiwi is *A. arguta* var. *arguta*. The name is common for the three previously described varieties: *A. arguta* var. *purpuea* (Rehder) C.F. Liang, *A. arguta* var. *cordifolia* (Miq.) Bean, and *A. arguta* var. *nervosa* C.F. Liang. *A. arguta* var. *giraldii* (Diels) Vorosch is a less common variety originating from the Sichuan province of China [9,10].

*A. arguta* is native to East Asia, from the Russian Far East, China and Korea to Japan and Malaysia, where it grows mainly in luminous forests. Additionally, *A. arguta* in its wild state spreads across the Northeastern United States as an invasive plant forming dense monocultures, which can be surprising [11].

Although the genus *Actinidia* was created by Lindley as early as 1836 [9], the currently valid taxonomy was established in 2007 [12]. Accordingly, it comprises 55 species (total of 76 taxa including 21 subspecies), 44 of which are endemic to China. The genus is divided into four sections: Stellatae, Strigosae, Maculatae, and Leiocarpae. *A. arguta* belongs to the last one, the most resistant in terms of frost hardiness. Leiocarpae includes the other frost-resistant *Actinidia* species: *A. kolomikta* (Maxim. & Rupr.) Maxim., *A. polygama* (Siebold and Zucc.) Maxim., *A. melanandra* Franch., *A. macrosperma* C.F. Liang, and *A. hyperleuca* Nakai. The skin of their fruits is smooth, hairless, and spotless [13].

For clarity, from this point the name *A. arguta* will be used, which de facto means *A. arguta* var. *arguta* (full scientific name *Actinidia arguta* (Siebold & Zuccarini) Planchon ex Miquel). Similarly, *A. deliciosa* will be used for *A. chinensis* var. *deliciosa* and *A. chinensis* for A. *chinensis* var. *chinensis*. In the non-specialist literature and colloquially, such common names as arctic kiwi, baby kiwi, bower *Actinidia*, bower vine, cocktail kiwi, dessert kiwi, hardy kiwi, Japanese Actinidia, kiwiberry, mini kiwi, Siberian gooseberry, taravine, and vinepear are used for *A. arguta* [14]. In this paper the names hardy kiwi, kiwiberry and mini kiwi are used interchangeably with *A. arguta.*

Although *A. arguta* is native to Eastern Asia, it is widely cultivated in many parts of the world, including Europe, North and South America, as well as New Zealand because of its hardiness, which allows it to withstand a wide range of temperatures including extremely low ones (−32 °C) for some cultivars. However, to acclimate to the cold and survive, they require gradual drops in temperature. Currently, there are many cultivars of *A. arguta* available, each with different sensory characteristics. The well-known ones are the following: ‘Ananasnaya’, ‘Geneva’, ‘Weiki’, ‘Issai’, ‘Jumbo’, ‘Ken’s Red’, and ‘Maki’ [15,16].

*A. arguta* differs from *A. chinensis* or *A. deliciosa* in certain characteristics, including a smaller size of the fruit and smooth, hairless, edible skin [17]. The fruit is usually eaten raw as a mini snack. It can also be remade into jams, wines, and other edibles. It should be emphasized that not only are the fruits rich in bioactive ingredients with multiple pharmacological and therapeutic effects, but the leaves, roots and stems are as well. The flowers are a source of essential oils [18]. *A. arguta* is distinguished by its exceptionally large content of biologically active compounds that positively affect the human body. It is one of the richest sources of vitamin C and many other biologically active compounds [10]. Due to its large content of vital nutrients (about 20), valuable to the human body, mini kiwi is considered a “healthy food” or “superfood.” According to the literature reports [19], over 500 compounds were isolated from the plant, which can be potentially used as ingredients for food, cosmetic and drug industries [18].

## 2. Bioactive Compounds of *Actinidia arguta* Fruits

### 2.1. Organic Acids, Polyphenols, Volatiles, Pigments

Besides being the well-known antioxidant product, mini kiwi is characterized by anti-inflammatory, anticancer, antidiabetic and antidermatitic properties as well as neuro-, nephro-, hepato- and cardioprotective actions. Most of these properties are related to the large content of polyphenolic compounds, which are considered powerful antioxidants. Polyphenols can be divided into the following [20]:

- Phenolic acids, e.g., caffeic or chlorogenic acid.
- Flavonoid polyphenols like catechin, epicatechin, quercetin and procyanidins.

Vitamin C, found in large quantities in *A. arguta*, also has strong antioxidant properties. It is associated with neuroprotective effects as well as anti-inflammatory and anticancer properties of mini kiwi. Antioxidant capacity, as well as the corresponding polyphenol and/or vitamin C content, is usually determined using spectrophotometric methods such as Folin–Ciocalteu for polyphenols or DPPH, ABTS, FRAP and ORAC methods to determine the total antioxidant capacity [21]. In order to determine the composition and to identify individual bioactive compounds, chromatography is used, mainly HPLC combined with spectroscopic detection methods (UV–VIS, DAD, MS) [22], while, in the case of volatile compounds, gas chromatography (FID, MS) can be applied [23]. As it was stated before, there is significant diversity of bioactive compounds discovered in *A. arguta*. Therefore, Table 1 presents only selected examples of analysis of bioactive compounds found in *A. arguta* fruits. These compounds are divided into four main classes: organic acids, phenolic acids, flavonoids and volatile compounds. There is also some information on pigments, like carotenoids and chlorophylls. The table also shows the sample preparation methods. It should be noted that both properties and flavor of the fruits depend largely on the contents and proportions of bioactive ingredients. The variability of their composition [24] is related not only to variety or cultivar but also to cultivation method and climate [25].

The most reliable information, regarding the variation in the contents depending on the variety/cultivar, concerns the largest group of bioactive compounds, i.e., polyphenols. According to Wojdyło et al. [33], who examined seven commercially available cultivars, the total phenolic content ranges from 2443.3 mg/100 g DM (for the ‘Jumbo’) to as much as 6679.18 mg/100 g DM (for the ‘Ananasnaja’), with the largest share of polymeric procyanidins, the content of which ranges from 2424 mg/100 g DM (for the ‘Jumbo’) to 6580 mg/100 g DM (for the ‘Ananasnaja’). The total flavonol content ranges from 9.53 mg/100 g DM (for the ‘Jumbo’) to 72.05 mg/100 g DM (for the ‘Ananasnaja’) and up to 108.45 mg/100 g DM (for the ‘Bingo’). Even smaller contents were observed for phenolic acids, the total content of which ranges from 8.88 mg/100 g DM (for the ‘Jumbo’) to approximately 24.5 mg/100 g DM (‘Ananasnaja’ and ‘Geneva’) cultivars. As it was stated before, the content of bioactive compounds depends not only on cultivars but also place and method of cultivation as well as storage conditions and post-harvest handling [24,25]. However, there are not enough consistent and comprehensive data concerning influence of these factors on the content of bioactive compounds.

### 2.2. Vitamin C

Vitamin C is one of the most important water-soluble vitamins, a crucial component of the human diet, commonly found in fruits and vegetables [35]. In addition to neutralizing free radicals [36,37] and reducing the incidence of heart disease and cancer [38], it participates in muscle, bone, and teeth construction [39], and it supports the absorption of iron and amino acids from food as well as prevents scurvy. The daily requirement for vitamin C can be satisfied by eating one kiwi fruit [31]. The literature proved that vitamin C is one of the main components determining the antioxidant properties of *Actinidia* fruits [16], including *A. arguta* [40,41]. With this in mind, this paper devotes a separate section to vitamin C, and Table 2 summarizes the data on the studies on the vitamin C content in mini kiwi.

As the data indicate, the vitamin C content of mini kiwi fruit is extremely large (up to 300 mg/100 g FW), much larger than that of kiwi fruit (60–120 mg/100 g) and comparable to wild rose (250–800 mg/100 g), blackcurrants (150–300 mg/100 g) and sea buckthorn (200–600 mg/100 g) [47].

The variability declared by researchers results undoubtedly from the genetic differences, cultivation and storage conditions as well as the method of sample preparation.

As already stated the method of storing the fruit, including the temperature, is an important factor influencing the levels of active compounds, such as polyphenols and vitamin C, in *A. arguta*. For example, when stored at 1 °C, the ascorbic acid content initially increases and then drops rapidly. This process occurs much more slowly under ultra-low oxygen conditions (1.5% CO_2_ and 1.5% O_2_). Similar changes are observed for other compounds. Total phenolic content decreases drastically when the fruit is stored at room temperature (22 °C). Although their content fluctuates during storage, lowering the temperature helps to inhibit this process. The same applies to flavonoids, whose content remains fairly constant at 1 °C [45].

## 3. Biological and Health-Promoting Properties of *A. arguta* Fruits

As it was illustrated by the tables above, mini kiwi is an excellent source of bioactive compounds that is characterized by high levels of nutritional value. They give it unique health-promoting properties, indicating that mini kiwi should be a permanent part of the human diet [48].

The most valuable biological properties are briefly summarized below.

### 3.1. Antioxidant Activity

The antioxidant properties of compounds or mixtures are primarily related to their ability to protect the body against the negative effects of reactive oxygen and nitrogen species [49,50]. This is directly related to protecting the body against oxidative stress, which has serious consequences for the living organism. There are various analytical techniques available for determining the antioxidant activity of biological samples, including food and plant extracts. The research on antioxidant properties can be generally divided into a-cellular and cellular assays.

#### 3.1.1. A-Cellular Assays

Common methods in this group are spectrophotometric ones, in which antioxidant activity is a measure of a sample’s ability to neutralize radicals (such as the ABTS or DPPH methods), protection against oxidation of another molecule (e.g., ORAC, beta-carotene method), or the ability to reduce metal ions (e.g., FRAP or CUPRAC) [21]. According to the literature, extracts from mini kiwi fruit have been extensively studied, and antioxidant activity has been confirmed using the aforementioned methods. The paper [28] demonstrated that the extracts from the *A. arguta* fruits exhibit antioxidant properties (assessed using the DPPH method) weaker compared to those of *A. kolomikta*, *A. purpurea*, *A. melanandra* and *A. macrosperma* but slightly better than *A. deliciosa*. In the paper [22], the antioxidant properties of extracts obtained from various *A. arguta* cultivars were determined using four complementary tests: ABTS, DPPH, FRAP, and CUPRAC. Although the results obtained using these four methods varied, they indicated a clear relationship between the polyphenol content and the antioxidant activity. The cultivars with the highest polyphenol content (‘Bidan’, ‘M1’, ‘Bingo’, ‘Anna’, and ‘Geneva’) exhibited the highest antioxidant activity. According to the paper [30], the hydroxyl radical scavenging capacity of mini kiwifruit proved to be much larger (5143.67 μmol VCE/100 g dry weight) than that of green kiwi berries (*A. delicosa*) (55.58 μmol VCE/100 g dry weight) as well as larger than that of apples (309.2 μmol VCE/100 g dry weight) and cranberries (1019.9 μmol VCE/100 g), where VCE is Vitamin C Equivalent. Furthermore, some *Actinidia* species, such as *A. arguta*, exhibited a larger antioxidant capacity, assessed using the FRAP method, compared to such vegetables as peppers, tomatoes, and spinach [51]. In all studied cases, differences in the antioxidant capacity of the studied *Actinidia* species, including *A. arguta*, can be tentatively attributed to different contents of polyphenols and ascorbic acid. Moreover, the antioxidant activity of fruits changes with the ripening time. Figiel-Kroczyńska et al. [52] found that the largest antioxidant properties (measured by the free radicals DPPH^•^, ABTS^•+^, and iron reduction (FRAP) were exhibited increasingly with the content of anthocyanins and consequently with the intensity of the red color of the skin of ‘Issai’ and ‘Ken’s Red’ cultivars. Essential oils (AEOs) obtained from *A. arguta* fruits also exhibit antioxidant properties. The studies carried out by Wang et al. [34] showed that AEO exhibited the concentration-dependent DPPH radical scavenging activity (IC_50_ = 117.60 μg/mL), hydroxyl radical scavenging activity (IC_50_ = 35.15 μg/mL), and the ability to protect beta-carotene using the beta-carotene method (IC_50_ = 73.60 μg/mL). Unfortunately, in each case, the determined values were much lower than those for the standard antioxidants (BHT and BHA). The latter yielded much lower IC_50_ values, indicating larger antioxidant properties than the tested oils.

#### 3.1.2. Cellular Assays

One of the methods for assessing the antioxidant activity is the Cellular Antioxidant Activity Assay (CAA). The active ingredients from the extracts are absorbed into cells, where they react with ROS intracellularly or by blocking peroxyl radicals generated at the cell membrane [30]. As follows from the papers [30,53], the CAA values for mini kiwifruit (72.83–250.78 μmol QE/100 g dry weight) were higher than for kiwifruit (55.68–121.72 μmol QE/100 g dry weight). This indicates that mini kiwifruit is a better source of antioxidants at the cellular level than kiwifruit.

### 3.2. Neuroprotective Properties

The neuroprotective effects of *A. arguta* in mice with the 1-methyl-4-phenyl-1,2,3,6-tetrahydropipridine (MPTP)-induced Parkinson’s disease model were investigated by Kitamura et al. [54], who administered *A. arguta* juice to 7-week-old mice continuously for 10 days before the first MPTP injection. Degeneration of dopaminergic neurons in the substantia nigra was induced with MPTP (30 mg/kg, intraperitoneally) once daily for five consecutive days. Based on this study, *A. arguta* ameliorates motor impairment and inhibits neuronal decline. The authors suggested that *A. arguta* can provide neuroprotection, thereby delaying or preventing the neurodegenerative process in Parkinson’s disease.

The ethyl acetate extract obtained from *A. arguta* fruit demonstrated protective and ameliorative effects on beta-amyloid (Aβ)-induced neurotoxicity and cognitive deficits in mice [55]. Furthermore, the extract enhanced the cholinergic system and inhibited the decline in the biochemical antioxidant activity in the mouse brain tissue. It also prevents mitochondrial dysfunction, which is associated with the inhibition of proapoptotic proteins. Based on these findings, this study suggests that the examined extract can be a potential substance for preventing Alzheimer’s disease by improving cognitive function [55,56]. In a study carried out by Jeong et al. 2020 [32], three mini kiwifruit cultivars (cv. ‘Mansu’ (*A. arguta* × *A. deliciosa*), cv. ‘Haeyeon’ (*A. arguta*), and cv. ‘Chiak’ (*A. arguta*) were proven to have a protective effect on the neuronal PC-12 and SHSY5Y cells exposed to hydrogen peroxide by increasing cell viability and reducing intracellular oxidative stress.

### 3.3. Anticholinergic Activity (In Vitro)

The human brain contains two main forms of cholinesterase, acetylcholinesterase (AChE) and butyrylcholinesterase (BChE) [57], which interrupt neurotransmission by hydrolyzing the neurotransmitter acetylcholine in neuronal cells. Cholinesterase inhibitors can improve neurotransmission by maintaining normal acetylcholine levels in cholinergic synapses. Acetylcholine plays a significant role in the learning process; its loss impairs memory in patients with Alzheimer’s disease. Therefore, inhibiting cholinesterase activity is a fundamental treatment strategy for this neurodegenerative disease [58]. Three cultivars of mini kiwi were proven to have cholinesterase-inhibiting effects, suggesting that they may be an effective source of inhibitors in the human diet [32]. The extracts from the ‘Mansu’, ‘Haeyeon’, and ‘Chiak’ cultivars at a concentration of 400 μg/mL exhibited moderate AChE inhibitory activity, approximately 9.7%, 7.1%, and 6.0%, respectively, while their BChE inhibitory activity was approximately 6.1%, 3.4%, and 4.1%. The ‘Mansu’ cultivar exhibited larger AChE and BChE inhibitory activity than the other cultivars used in this study. The cholinesterase inhibitory activity of *A. arguta* ‘Geneva’ cultivar fruits was investigated by Wojdyło et al. [29]. In these studies, the percentage of AChE and BChE inhibition was 79.7 and 93.9, respectively. The results obtained for these enzymes were similar and correlated well with the total phenolic content. Among the polyphenols with the greatest ability to inhibit AChE were quercetin, kaempferol, myricetin, and phloretin, while the most effective BChE inhibitors were apigenin, luteolin, quercetin, phloretin, and cyanidin [59]. According to Sawicki et al. [60], the content of (+)-catechin and L-ascorbic acid in *A. arguta* fruit correlated with the anti-AChE parameter. The cholinesterase inhibitory potential of ‘Weiki’ cultivar fruits was assessed in untreated and high hydrostatic pressure-treated (450, 550 or 650 MPa for 5 or 15 min) samples. The study demonstrated that exposing the ‘Weiki’ cultivar to high pressure significantly improved the AChE inhibition. However, this increase is likely related to the increased concentration of certain polyphenols resulting from the processing [61].

### 3.4. Antitumor

The in vitro studies on the anticancer role of *A. arguta* fruit juice proved that it reduces methylnitrosamino)-1-(3-pyridyl)-1-butanone-induced lung carcinogenesis. The juice components, including isoquercetin, affect both the initiation and growth/progression stages of carcinogenesis through antimutagenesis, stimulation of alkyl-DNA adduct repair, and suppression of AKT-mediated growth signaling [62]. The antimutagenic effect of hardy kiwi juice was also demonstrated in the studies [63], in which mini kiwi juice inhibited the mutagenicity of heterocyclic amines (MeIQx, Trp-P-2, and PhIP), aflatoxin B1 and polycyclic aromatic hydrocarbons (benzo(a)pyrene and DMBA). Furthermore, it was proven that the mini kiwi juice inhibited formation of DNA adducts in the liver of mice whose diet contained deliberately tumor-initiating ingredients. It was confirmed that the juice components led to reduction in the number of existing skin tumors in mice [63]. Relatively well-studied anticancer components of *A. arguta* are its polysaccharides, which inhibit proliferation of Hep G2 cells by blocking the cell cycle and inducing apoptosis. The results of this study demonstrate that *A. arguta* polysaccharides can be a potential source of natural anticancer agents and could be used as a natural product with anticancer activity [64]. Mini kiwi, as a source of many active components (rutin, kaempferol, ferulic acid, vitamin C, vitamin E, etc.), has a potential impact on the glioblastoma multiforme. It demonstrates an inhibitory effect on cancer cell self-renewal, modulates signaling pathways involved in regulating cell phenotype and metabolism, and influences the consolidation of the tumor microenvironment [65].

### 3.5. Anti-Inflammatory Properties

The inflammatory process is the body’s crucial defense mechanism against pathogens (viruses or bacteria). However, chronic inflammation caused by the constant activation of macrophages can lead to serious adverse effects, like autoimmune diseases and cancer. Therefore, substances that can protect the body against inflammation, especially chronic inflammation, are being sought [66]. Numerous studies proved that the extracts from *A. arguta* fruit exhibit anti-inflammatory properties [67,68]. The anti-inflammatory effects of kiwiberry extracts were analyzed using the RAW 264.7 cells (macrophage-like cells derived from mice and widely used in studies of the immune system, inflammation, metabolism, and bone biology), in which inflammation was induced by LPS (a component of the cell wall of Gram-negative bacteria that acts as an endotoxin). The anti-inflammatory properties of the extracts were determined by measuring the levels of TNF-α and IL-6 (proinflammatory cytokines produced by the body) in the cell culture media. Inhibiting the pathway leading to the production of these cytokines can be a potential mechanism of anti-inflammatory activity. The authors found that the mini kiwi extracts significantly reduced the release of IL-6 and TNF-α in a dose-dependent manner [67]. In the paper, an aqueous extract derived from the fruit of *A. arguta* has been studied as an anti-inflammatory agent in humans and atopy-like animal models. The study of allergy-related cytokines from the peripheral blood mononuclear cells (PBMCs) stimulated with the house dust mite allergen (DME) extract in dogs following the treatment with the *A. arguta* extract demonstrated effective inhibition of the inflammatory cytokines TNF-α, IL-4, IL-5, IL-13, and IFN-γ [68].

### 3.6. Antidiabetic Activity

Type 2 diabetes is considered a component of metabolic syndrome, defined as abnormal glucose tolerance with impaired glucose and lipid metabolism caused by disturbed insulin secretion or insulin resistance. One effective treatment for diabetes is inhibiting postprandial hyperglycemia by inhibiting or delaying the absorption of carbohydrates from meals. In the human body, dietary carbohydrates are hydrolyzed by pancreatic α-amylase and intestinal α-glucosidase, the enzymes responsible for the breakdown of oligosaccharides and disaccharides into digestible monosaccharides. To inhibit the postprandial hyperglycemia, inhibitors of these enzymes should be used [69].

The research carried out by Wojdyło et al. [29,33] confirmed that the *A. arguta* extracts are good inhibitors of α-amylase and α-glucosidase. Their efficacy was larger than that of the *A. arguta* × *A. purpurea* and *A. arguta* × *A. melanandra* extracts. The inhibitory activity of glucosidase increased from ‘Geneva’ (IC_50_ = 0.99) to ‘Weiki’ (IC_50_ = 0.42), while for α-amylase it increased from ‘Weiki’ through ‘Geneva’ and ‘Ananasnaja’ to ‘Jumbo’. This is consistent with the results obtained in our laboratory using the TLC-bioassays, in which 10 cultivars of *A. arguta* were compared in terms of their enzyme-inhibitory properties [70]. Furthermore, the results indicate that the tested *A. arguta* cultivars exhibited stronger activity than *A. deliciosa*.

### 3.7. Inhibitory Effect on Digestive Enzymes

One of the most important enzymes responsible for triglyceride digestion is pancreatic lipase. Inhibiting this enzyme is one way to inhibit or delay the digestion and absorption of triglycerides. The studies by Li et al. [31] demonstrated that the extracts obtained from various kiwifruit cultivars, including mini kiwifruit, exhibit varying levels of pancreatic lipase inhibitory activity. The IC_50_ values for the inhibitory effect on pancreatic lipase ranged from 3.12 ± 0.09 mg/mL to 7.44 ± 0.11 mg/mL among the tested kiwifruit. The authors attribute the obtained value differences to the varying contents of polyphenolic compounds, primarily proanthocyanidins and quercetin derivatives [71,72]. The best properties (and the lowest IC_50_ value) were obtained for *A. chinensis* cv. ‘Hongshi’ and the poorest for *A. macrosperma*. Among the tested extracts, *A. arguta* extract demonstrated relatively small lipase-inhibiting properties compared to the other tested extracts (IC_50_ around 6 mg/mL) but comparable to the commercially available drug orlistat (IC_50_ = 6.34 mg/mL). In turn, the studies carried out by Qiao et al. [73] on the extracts of kiwi berry (*A. arguta* (Sieb & Zucc) Planch. ex Miq. cv. ‘Longcheng No. 2’) demonstrated that the crude kiwi fruit extract exhibited a lipase-inhibiting activity, with an IC_50_ value of 21.13 mg/mL. This value was compared to that obtained for black chokeberry (IC_50_ = 83.45 mg/mL), allowing the authors to conclude that the mini kiwi fruit possesses a lipase-inhibiting potential.

### 3.8. Antiatherosclerotic Activity

Atherosclerosis is considered a multifactorial disease in which hyperlipidemia plays a significant role. It has long been believed that a proper diet, particularly one rich in fruits and vegetables, has a significant impact on the course of this disease as well as other cardiovascular diseases [74]. Atherosclerotic changes were found in experimental animals (rats, mice, and rabbits) when their diet was supplemented with cholesterol [75]. Leontowicz et al. [75] proved that a diet rich in various cultivars of mini kiwi fruit increased the antioxidant activity of blood plasma in rats, significantly reducing LDL levels and improving all lipid parameters. Thus, the studies confirmed that the mini kiwi fruits exhibit hypolipidemic and hypocholesterolemic effects. Furthermore, their anti-inflammatory properties protect the aortas and livers of rats loaded with exogenous cholesterol. As follows from the more detailed studies, dietary supplementation with the mini kiwifruit, particularly the ‘Geneva’ cultivar, reduces liver fat accumulation in hypercholesterolemic rats, likely by reducing the expression of key genes involved in cholesterol metabolism. Therefore, the mini kiwifruit could be used as a potential dietary component for cholesterol control [76]. This was confirmed by a pilot study carried out on 43 volunteers, which showed that, in humans, mini kiwi supplementation caused changes in metabolic parameters and a significant decrease in triglyceride levels [77].

### 3.9. Antimicrobial Properties

According to the study by Wang et al. [34], essential oil obtained from *A. arguta* fruit demonstrated a significant antimicrobial activity against *Staphylococcus aureus* and *Saccharomyces cerevisiae* but showed moderate effectiveness against *Bacillus subtilis* and *Microsporum canis*. However, the Gram-negative bacteria, *Escherichia coli* and *Pseudomonas aeruginosa*, were not susceptible to the essential oils. The antibacterial activity of the fruit extracts from *A. arguta* cv. ‘Veresneva’ against the Gram-positive and Gram-negative strains was studied by Khromykh et al. [78]. The obtained results confirmed the known large bioactivity of *Actinidia* plants. The *A. arguta* fruit extracts demonstrated the largest inhibitory activity against the Gram-negative strains, such as *E. coli B906* and *P. aeruginosa B907*, followed by the Gram-positive strains *Micrococcus lysodeikticus 2665* and *S. aureus B904*. In the paper [79], the *A. arguta* fruit extract obtained under the optimized conditions of ultrasound-assisted extraction demonstrated a large antimicrobial activity against *S. aureus* (MIC = 32 mg/mL) and *Porphyromonas gingivalis* (MIC = 64 mg/mL). Additionally, it reduced the growth rate of *E. coli*. It should be noted that the antimicrobial activity also depended on the type of solvent (selectivity) used to prepare the extract as well as on the cultivation conditions and harvest date [80]. Generally, the study proved that methanol extracts from the kiwifruit demonstrated stronger antimicrobial activity compared to the ethanol extracts. The methanol extracts from the kiwifruit collected in 2017 demonstrated the strongest, taking into account the tested ones, antimicrobial activity against *Vibrio parahemolyticus*, *Enterococcus faecalis*, *Salmonella typhimurium*, and *Proteus vulgaris* (12.5 ± 0.7 mm). The ethanol extracts from kiwifruit (40 μL) collected in 2018 also demonstrated the largest antimicrobial activity against *Listeria monocytogenes* (13.5 ± 0.7 mm).

### 3.10. Antiallergic Activity

The antiallergic effect of the extract obtained from the *A. arguta* fruit in a mouse asthma model was investigated in the paper by Kim et al. [81]. To investigate the effect on asthma, mice were orally administered some substances to induce asthma symptoms. Airway hyperresponsiveness (AHR), bronchoalveolar lavage fluid, serum, and lung tissue were analyzed using various methods. The study found that the extract could alleviate asthma symptoms, including AHR and eosinophilia in the lungs. Making asthma symptoms more evident was accompanied by reduction in the IL-5 and IgE levels and an increase in the expression of heme oxidase-1 (potent anti-inflammatory enzyme in the alveolar inflammatory cells), as well as an increase in the mRNA levels of foxp3, TGF-b1, and IL-10, important markers of regulatory T cells, in lung tissue. Thus, the extract from the edible fruits of *A. arguta* controls various factors associated with the pathogenesis of allergy. Subsequent studies proved that the activity of the extract can be reproduced by mixing the chosen compounds present in them [82]. Individually or as mixtures, six chemical compounds (citric, quinic, and malic acids, myoinositol, isoquercitin, and 5-hydroxymethyl-2-furaldehyde) were tested for their effects on the expression of various Th2 cytokines and inflammatory mediators in the cell-based assay. The mixtures reproduced the original activities of the extract to a significant extent. The studies proved that a significant antiallergic activity can be reproduced, both in vitro and in vivo. The six identified compounds will enable the future development of a new type of antiallergic drug.

Antiallergic effects were also observed in fatty acids isolated from the *A. arguta* fruits. The studies show that various compounds, such as linoleic acid, α-linolenic acid, ethyl linoleate, ethyl linolenate, and ethyl stearate, isolated from the mini kiwi fruits, have potential as inhibitors of IL-4 production, which correlates with the occurrence of allergic diseases [83].

For the sake of scientific accuracy, it should be mentioned here that mini kiwi can also cause allergies, which is mainly due to a large content of the protein actinidin (cysteine protease) [84].

## 4. Review Methodology

The presented literature review was conducted by collecting, reviewing, and collating information (from 2003 to 2025, with a large preponderance of papers from the last 10 years) from available online databases, such as Google Scholar, Scopus, Web of Science, PubMed, and Science Direct. The search was made using keywords (“biological properties of *Actinidia arguta*,” “active compounds of *Actinidia arguta*,” “*Actinidia arguta* occurrence,” and “*Actinidia arguta* analysis”). The search was limited to papers in English. Furthermore, the abstracts were prescreened before the full text was analyzed. The literature review was thoroughly analyzed to summarize the general knowledge on *A. arguta*. The search results were reviewed individually by two authors.

## 5. Conclusions

*A. arguta* fruits are becoming increasingly common on the global consumer market, although they are not as common as *A. deliciosa* or *A. chinensis*. However, as the attempt was made to show, more and more publications appear on the subject of mini kiwi: its chemical composition, biologically active substances and health-promoting properties. Many papers also discuss the differences between various cultivars of *A. arguta*.

Our paper concerns fruits, which are particularly important from the consumer’s point of view. We have only mentioned that the subject of research should also include leaves, stems, and flowers, which can become a source of biologically active substances for the cosmetic, food and pharmaceutical industries. In particular, leaves, which are a rich source of polyphenols, are increasingly attracting the interest of researchers [85].

Our article focuses on *A. arguta* fruit analysis and highlights its rich bioactive components, which, in addition to its flavor, are responsible for its health-promoting properties. By putting greater emphasis on these properties and educating potential consumers about their benefits, it is expected that more and more people will appreciate and enjoy the taste of these small, inconspicuous fruits. The information contained in this review paper should contribute to the development of further research on *A. arguta*, which will likely lead to the isolation and identification of additional components in the future. Furthermore, undoubtedly, new research will result in a better and more effective understanding of pharmacological effects of mini kiwi, which will ultimately benefit humanity and contribute to the advancement of medical science.

## Figures and Tables

**Table 1:** Examples of the *A. arguta* fruit analysis.

Chemical Composition	Major Compounds	Sample Preparation	Methods of Determination	References
Organic acids	oxalic acid quinic acid malic acid shikimic acid lactic acid citric acid	not specified	HPLC-UV/VIS	[26]
	oxalic acid quinic acid malic acid shikimic acid lactic acid citric acid	not specified	not specified	[27]
Phenolic acids	tannic acid, 2,5-dihydroxybenzoic acid, hydroxybenzoic acid, chlorogenic acid, caffeic acid	extraction of homogenized fresh fruit with acidified methanol concentration (methanol evaporation)	HPLC-UV/VIS	[28]
	caffeic acid-*O*-hexoside neochlorogenic acid, chlorogenic acid, trans–p-coumaryoyl quinic acid	centrifugation of freeze-dried fruit with a mixture of methanol/water/acetic acid/ascorbic acid (30:68:1:1, *v*/*v*) sonication of the mixture	LC-MS-PDA-Q-TOF (for identification) UPLC-PDA (for quantification)	[29]
	protocatechuic acid caffeic acid chlorogenic acid	extraction of freeze-dried fruit with methanol	UPLC-Q-TOF-MS (for identification) HPLC-PDA-MS (for quantification)	[18]
	gallic acid chlorogenic acid caffeic acid coumaric acid	extraction of fresh fruit with methanol and 2,6-di-tert-butyl-4-methylphenol filtration	HPLC-UV/VIS	[30]
	chlorogenic acid neochlorogenic acid caffeic acid	extraction of freeze-dried fruit with acidified methanol sonication of the mixture centrifugation concentration (by evaporating methanol)	HPLC-DAD	[31]
	neochlorogenic acid cryptochlorogenic acid	extraction of fresh fruit with aqueous ethanol centrifugation concentration (by the solvent evaporation)	HPLC-DAD	[32]
	caffeic acid-O-hexoside neochlorogenic acid chlorogenic acid cryptchlorogenic acid	extraction of freeze-dried fruit with acidified methanol sonication of the mixture centrifugation filtration	LC-PDA/MS QTof UPLC-PDA-FL	[33]
Flavonoids	Flavonols (quercetin) Flavanols ((+)-catechin, epicatechin)	extraction of homogenized fresh fruit with acidified methanol centrifugation filtration concentration (methanol evaporation)	HPLC-UV/VIS	[28]
	Flavonols (quercetin-3-*O*-rutinoside, quercetin-3-*O*-glucoside, quercetin-3-*O*-(acetyl-rhamnoside)-(1⟶6) galactoside, keampferol-3-*O*-galactoside, keampferol-3-*O*-glucoside) Flavanols (proanthocyanidin B_1_, proanthocyanidin B_3_, proanthocyanidin B_4_, ((+)-catechin, epicatechin, procyanidin C_1_, proanthocyanidin dimer) Anthocyanins (cyanidin-3-*O*-samubioside)	extraction of freeze-dried fruit with a mixture of methanol/water/acetic acid/ascorbic acid centrifugation filtration	LC-MS-PDA-Q-TOF (for identification) UPLC-PDA (for quantification)	[29]
	Flavonols (quercetin-3-*O*-rutinoside, quercetin-3-*O*-galactoside, quercetin-3-*O*-glucoside) Flavanols ((+)-gallocatechin, Proanthocyanidin B_2_, Proanthocyanidin C_1_,)	extraction of freeze-dried fruit with methanol	UPLC-Q-TOF-MS (for identification) HPLC-PDA-MS (for quantification)	[18]
	Flavonols (quercetin, quercetin-3-*O*-rutinoside) Flavanols ((+)-catechin, (−)-epicatechin,)	extraction of fresh fruit with methanol and 2,6-di-tert-butyl-4-methylphenol filtration	HPLC-UV/VIS	[30]
	Flavonols (quercetin, quercetin-3-*O*-rutinoside, quercetin-3-rhamnoside) Flavanols ((+)-catechin, procyanidin B_1_; procyanidin B_2_)	extraction of freeze-dried fruit with acidified methanol sonication of the extract centrifugation concentration (methanol evaporation)	HPLC-DAD	[31]
	Flavonols (quercetin-3-*O*-rutinoside, isoquercitrin, hyperoside) Flavanols (procyanidin B_2_, epicatechin)	extraction of fresh fruit with aqueous ethanol centrifugation concentration (ethanol evaporation)	HPLC-DAD	[32]
	Flavonols (quercetin-3-O-galactoside, quercetin-3-O-rutinoside, quercetin-3-O-glucoside, quercetin-3-O-(acetyl-rhamnoside)-(1⟶6) -galactoside, keampferol-3-O-galactoside Flavanols (polymeric procyanidins Anthocyanins (cyanidin-3-*O*-samubioside)	extraction of freeze-dried fruit with acidified methanol sonication of the extract centrifugation filtration	LC-PDA/MS Q-TOF UPLC-PDA-FL	[33]
Volatile Compounds	Terpenes (camphor, eucalyptol, α-terpineol, terpinolene) Benzenoid compounds (ethyl benzoate, methyl benzoate)) Esters (ethyl butanoate, ethyl hexadecanoate, ethyl hexadec-9-enoate, ethyl linoleate, ethyl linolenate, ethyl octanoate, 1-methylethyl tetradecanoate, methyl hexadecanoate, methyl linoleate, methyl oleate) Aldehydes (hepta-E2,E4-dienal, hexanal, hex-E2-enal) Ketones (octan-2,3-dione, pent-E3-en-2-one) Alcohols (dodecanol, hexanol) Hydrocarbons (2-methylpenta-1,3-diene, octadecane) Sulfur compounds (bis(1-methylethyl)disulphide)	head space (fruit pulp was placed in the headspace adapter with the magnetic stirrer solvent extraction (fruit pulp was extracted first with EtOH, then distilled to remove solids and waxes; the resulting distillate was mixed with pentane)	GC-FID GC-MS	[23]
	Terpenes (dipentene M, dipentene D, camphen, α-pinene, myrcene, terpinolene) Esters (methyl butanoate, ethyl acetate, butyl acetate, and ethyl hexanoate) Aldehydes (Heptanal D, Butanal M, Butanal D, Benzaldehyde, Isobutyraldehyde M) Ketones (2-Octanone, L(-)-Carvone, Isomenthone, 2-Hexanone 3-Hepten-2-one) Alcohols (Carveol D, hexanol, 3-Methyl-1-butanol, 1-Butanol) Pyrazines (2-Methoxy-3-methylpyrazine, 2-Methoxy-3-methylpyrazine, 2-Ethyl-3-methylpyrazine) Furans (2,5-Dimethylfuran, 2-Pentylfuran)	homogenization in the headspace vial internal standard addition (4-methyl-2-pentanol) the sample was injected after incubation at 60 °C for 15 min.	HS-GC-IMS	[26]
	Terpenes (α-phellandrene, α-pinene, myrcene and terpinene) Esters (ethyl butyrate, butyl isovalerate, butyl acetate, hexyl acetate, hexyl propanoate, isopentyl acetate, isobutyl acetate and methyl butyrate) Aldehydes ((E)-2-hexenal, (E)-2-octenal, (Z)-4-heptenal, 1-hexanal, 1-nonanal, valeraldehyde, valeraldehyde, 3-Methyl butanal and benzaldehyde) Ketones (1-penten-3-one and acetoin) Alcohols (1-octen-3-ol) Pyrazines (2,3,5-Trimethylpyrazine) Furans (2,5-Dimethylfuran)	homogenization in the headspace vial internal standard addition (4-methyl-2-pentanol)	HS-GC-IMS	[27]
	Terpenes (ß–amyrin, α-amyrin) Esters (Linoleic acid butyl ester) Aldehydes (hexanal, 2-hexenal) Hydrocarbons (docosane, triosane, tetracosane, pentacosane, hexacosane, heptacosane, octacosane, triacosane, nonacosane, hentriacontane, squalene) Alcohols (cholest-5-en-3-ol, stigmaste-5,22–dien-ol, hexacosanol, γ-sitosterol)	homogenization extraction with hexane filtration solvent evaporation	GC-MS	[34]
Pigments	Carotenoids (lutein, β-carotene) Chlorophylls (chlorophyll *a* and *b*)	extraction of homogenized fresh fruit with hexane/methanol/butylhydroxyanisole mixture centrifugation solvent evaporation reconstitution in the mobile phase	HPLC–electrochemical detector	[28]
		homogenization of freeze-dried fruit with acetone/petroleum ether–carotenoids homogenization of freeze-dried fruit with acetone-chlorophylls	HPLC–PDA	[22]
			Spectrophotometrically; detection at the following λ (nm): 661.6 (chlorophyll *a*), 644.8 (chlorophyll *b*)	

**Table 2:** Vitamin C content in *A. arguta* fruits.

Vitamin C	Content	Sample Preparation	Methods of Determination	References
Total value *	86.1 to 106.6 mg/100 g FW **	extraction of fresh fruit with metaphosphoric acid/perchloric acid mixture reduction of dehydroascorbic acid to ascorbic acid in the presence of dithiothreitol	HPLC-UV/VIS	[28]
Non-total value *	50 to 93.94/100 g FW	extraction of fresh fruit with metaphosphoric acid/perchloric acid mixture	HPLC-UV/VIS	[30]
Total value	37 to 185 mg/100 g FW	extraction of the fresh fruit homogenate with glacial 5% orthophosphoric acid containing EDTA. centrifugation of the blended mixture reduction of dehydroascorbic acid using tris[2-carboxyethyl]phosphine.	HPLC-UV/VIS	[42]
Total value	144.7–218.1 mg/100 g FW	extraction of fresh fruit with metaphosphoric acid/perchloric acid mixture purification reduction of dehydroascorbic acid to ascorbic acid in the presence of dithiothreitol	HPLC-UV/VIS	[43]
Non-total value	51.32 mg/100 g FW	homogenization of fresh fruit with 5%, *w*/*v* trichloroacetic acid centrifugation of the mixture mixing the supernatant with trichloroacetic acid, ethanol, phosphoric acid in ethanol, 1,10-phenanthroline, and iron(III) trichloride	Spectrophotometric measurements	[31]
Non-total value	55.2–130 mg/100 g FW	homogenization of fresh fruit with metaphosphoric acid (5%, *w*/*v*) separation using 5% (*w*/*v*) metaphosphoric acid solution adjusting the solution to a known volume with 5% (*w*/*v*) metaphosphoric acid solution three independent extractions were performed	HPLC-DAD	[32]
Non-total value	76.09–282.56 mg/100 g FW	homogenization of fresh fruit with 0.1 M metaphosphoric acid centrifugation and separation	LC-PDA/MS Q-Tof UPLC-PDA-FL	[33]
Total value	53.95 mg/100 g FW	extraction of fruit with oxalic acid reduction of dehydroascorbic acid to ascorbic acid in the presence of 2,6-dichlorophenoline-dophenol extraction of excess pigment with xylene	Spectrophotometric measurements	[44]
Non-total value	67.8–85.2 mg/100 g FW	extraction of fresh frozen fruit with metaphosphoric acid filtration	HPLC-UV/DAD	[45]
Total value	50–300 mg/100 g FW (depending on the cultivar and time after flowering)	extraction of fresh frozen fruit with metaphosphoric acid and acetic acid centrifugation, dilution and filtration reduction of dehydroascorbic acid to ascorbic acid in the presence of dithiothreitol solution (dissolved in the solution of potassium hydrogen phosphate)	HPLC-UV/VIS	[46]

* Total value (ascorbic + dehydroascorbic acid); Non total value (only ascorbic acid) ** FW-Fresh Product Weight.

## Data Availability

No new data were created or analyzed in this study. Data sharing is not applicable to this article.

## Abbreviations

The following abbreviations are used in this manuscript:

Aβ	beta-Amyloid
ABTS	2,2′-Azinobis(3-ethylbenzothiazoline-6-sulfonic acid)
AChE	Acetylcholinesterase
AEO	*Actinidia arguta* Essential Oil
AHR	Airway Hyperresponsiveness
BChE	Butyrylcholinesterase
BHA	Butylated Hydroxyanisole
BHT	Butylated Hydroxytoluene
CAA	Cellular Antioxidant Activity Assay
CUPRAC	Cupric Reducing Antioxidant Capacity
DAD	Diode Array Detector
DHA	L-dehydroascorbic acid
DMBA,	7,12-dimethylbenz(a)anthracene
DME	Dust Mite Allergen Extract
DNA	Deoxyribonucleic Acid
DPPH	2,2-diphenyl-1-picrylhydrazyl
DTT	Dithiothreitol
DW	Dry Weight
EDTA	Ethylenediaminetetraacetic Acid
FID	Flame Ionization Detector
FL	Fluorescence
Foxp3	Forkhead Transcription Factor
FRAP	Ferric Reducing Antioxidant Parameter
FW	Fresh Product Weight
HPLC	High-Performance Liquid Chromatography
HS	Headspace
IC_50_	Half-Maximal Inhibitory Concentration
IFN-γ	Interferon-Gamma
IgE	Immunoglobulin E
IL-4	Interleukin-4
IL-5	Interleukin-5
IL-6	Interleukin-6
IL-13	Interleukin-13
IMS	Ion Mobility Spectrometry
LDL	Low-Density Lipoprotein
LPS	Lipopolysaccharide
MeIQx	2-amino-3,8-dimethylimidazo[4,5-*f*]quinoxaline
MIC	Minimum Inhibitory Concentration
MPTP	1-methyl-4-phenyl-1,2,3,6-tetrahydropipridine
mRNA	Messenger Ribonucleic Acid
MS	Mass Spectrometry
ORAC	Oxygen Radical Absorbance Capacity
PBMCs	Peripheral Blood Mononuclear Cells
PDA	Photo Diode Array
PhIP	2-amino-1-methyl-6-phenylimidazo[4,5-*b*]pyridine
PTEF	Polytetrafluoroethylene
QE	Quercetin Equivalent
ROS	Reactive Oxygen Species
TCA	Trichloroacetic Acid
TGF-β1	Transforming Growth Factor beta 1
Th 2	Type 2 Lymphocyte
TLC	Thin-Layer Chromatography
TNF-α	Tumor Necrosis Factor α
TOF	Time of Flight
Trp-P-2	3-amino-1-methyl-5*H*-pyrido[4,3-*b*]indole
UPLC	Ultra-Performance Liquid Chromatography
UV	Ultraviolet
VIS	Visible
VCE	Vitamin C Equivalent
